# SIRT1 alleviates high-magnitude compression-induced senescence in nucleus pulposus cells via PINK1-dependent mitophagy

**DOI:** 10.18632/aging.103587

**Published:** 2020-07-18

**Authors:** Yiyang Wang, Haoming Wang, Yunyun Zhuo, Yanzhu Hu, Zetong Zhang, Jixing Ye, Liehua Liu, Lei Luo, Chen Zhao, Qiang Zhou, Pei Li

**Affiliations:** 1 Department of Orthopedics, The Third Affiliated Hospital of Chongqing Medical University, Chongqing 401120, China; 2 Tissue Repairing and Biotechnology Research Center, The Third Affiliated Hospital of Chongqing Medical University, Chongqing 401120, China; 3 Department of Orthopedics, Three Gorges Central Hospital, Chongqing 404000, China; 4 Department of Orthopedics, Southwest Hospital, Third Military Medical University, Army Medical University, Chongqing 400038, China

**Keywords:** SIRT1, compression, senescence, nucleus pulposus, mitophagy

## Abstract

Mechanical overloading-induced nucleus pulposus (NP) cells senescence plays an important role in the pathogenesis of intervertebral disc degeneration (IVDD). The silent mating type information regulator 2 homolog-1 (SIRT1)-mediated pathway preserves the normal NP cell phenotype and mitochondrial homeostasis under multiple stresses. We aimed to investigate the role of SIRT1 in IVDD by assessing the effects of SIRT1 overexpression on high-magnitude compression-induced senescence in NP cells. High-magnitude compression induced cellular senescence and mitochondrial dysfunction in human NP cells. Moreover, SIRT1 overexpression tended to alleviate NP cell senescence and mitochondrial dysfunction under compressive stress. Given the mitophagy-inducing property of SIRT1, activity of mitophagy was evaluated in NP cells to further demonstrate the underlying mechanism. The results showed that SIRT1-overexpression attenuated senescence and mitochondrial injury in NP cells subjected to high-magnitude compression. However, depletion of PINK1, a key mitophagic regulator, impaired mitophagy and blocked the protective role of SIRT1 against compression induced senescence in NP cells. In summary, these results suggest that SIRT1 plays a protective role in alleviating NP cell senescence and mitochondrial dysfunction under high-magnitude compression, the mechanism of which is associated with the regulation of PINK1-dependent mitophagy. Our findings may provide a potential therapeutic approach for IVDD treatment.

## INTRODUCTION

Intervertebral disc degeneration (IVDD) is one of the main causes of degenerative lumbar spine disease, which has major socioeconomic effects [1]. The specific pathomechanism involved in the process of IVDD is still unclear because it relates to both age-related changes and multiple stress-induced damage in intervertebral disc (IVD) tissues [2]. Hence, there is currently a lack of effective and long-lasting biotherapeutic strategies for IVDD. As an age-related pathology, the accumulation of senescent cells within the IVD tissue is common during the progression of IVDD [3, 4]. It has been confirmed that nucleus pulposus (NP) cells are responsible for the synthesis of the extracellular matrix (ECM) within the central region of the IVD, which first presents degenerative changes during IVDD [5]. Thus, one possible strategy for retarding the progression of IVDD is to alleviate senescence of NP cells under extrinsic stresses. Mechanical loading has been widely reported to play a critical role in the process of IVDD, and overloaded compression could accelerate the death or senescence of IVD cells and initiate disc degeneration [6–8]. Previously, we have conducted a series of studies to elucidate the role of different types of mechanical compression in regulating NP cell survival, and evaluated whether high-magnitude mechanical compression could substantially promote apoptosis- and senescence-like cellular fates of NP cells via multiple pathways [9–11]. Notably, consistent with our findings, numerous studies have reported that overloaded mechanical compression aggravated NP degeneration by inducing oxidative stress injury of NP cell [11–14]. Based on the free-radical theory of aging, oxidative stress is due to the accumulation of reactive oxygen species (ROS), which results in organelle dysfunction and triggers cellular senescence [15]. As mitochondria are the primary endogenous ROS-generating organelle, maintenance of the mitochondrial homeostasis has been widely considered as a key factor for reversal of cellular senescence [16, 17]. A previous study reported that high-magnitude compression profoundly exacerbates the apoptosis of NP cells by suppressing mitochondrial function and producing excessive ROS [18]. Furthermore, some mitochondrion-targeted compounds have been proven to protect against IVDD by ameliorating mitochondrial dysfunction and redox imbalance [13, 19]. Therefore, guarding the normal mitochondrial function to maintain redox homeostasis is seen as an effective way to alleviate the senescence of NP cells.

Silent mating type information regulator 2 homolog-1 (SIRT1), also known as Sirtuin 1, is a nicotinamide-dependent class 3 histone deacetylase that is essential for cell survival and increases the longevity of species ranging from yeast to mammals [20]. SIRT1 regulates multiple cellular processes, including the cell cycle, metabolism, and senescence [20–22]. Previous studies have reported that SIRT1 could inhibit the senescence and death of IVD cells by activating autophagy or deacetylating the P53/P21 and PI3K/Akt signaling pathways [23–26]. Therefore, it is conceivable that SIRT1 is a vital regulator of IVD cell survival via modulation of senescence. Additionally, recent studies have shown that SIRT1 plays the anti-senescence and antioxidant roles by regulating mitophagy [27–31]. Mitophagy is a type of mitochondria-specialized autophagy that can protect against cellular senescence by clearing injured mitochondria to maintain the redox homeostasis [32]. Several studies have reported that activation of mitophagy could promote the survival of NP cells and ameliorate the progression of IVDD by eliminating impaired mitochondria [33, 34]. In light of the antioxidant and mitophagy-related properties of SIRT1, we speculate that it may have beneficial effects on attenuating NP cell senescence by regulating mitophagy.

Here, the present study was conducted to assess the biological effect of SIRT1 on mitochondrial homeostasis and cellular senescence in human NP cells under high-magnitude compression and determine whether mitophagy participated in this process. For this study, human NP cells seeded in methacrylamide-modified gelatin (Gel-MA) scaffolds were perfusion-cultured and underwent mechanical compression using the substance exchanger-based perfusion bioreactor system. Cellular senescence was evaluated by analysis of senescence associated β-galactosidase (SA-β-Gal) activity and expression of senescence-related biomarkers. Mitochondrial function and mitophagy were detected by measuring the content of intracellular ROS, changes in mitochondrial membrane potential (ΔΨm), and expression of mitophagic markers and transmission electron microscopy (TEM) observations. The recombinant lentiviral vector construct containing SIRT1 (Ad-SIRT1) was used to upregulate SIRT1 expression to further verify the role of SIRT1 in NP cells under high-magnitude compression. Recombinant lentiviral vectors with short hairpin RNA targeting PINK1 (PINK1-shRNA) were used to inhibit mitophagy to test its role involved in the regulatory process.

## RESULTS

### SIRT1 expression decreased in NP tissues with intensified IVDD

**Figure 1:**
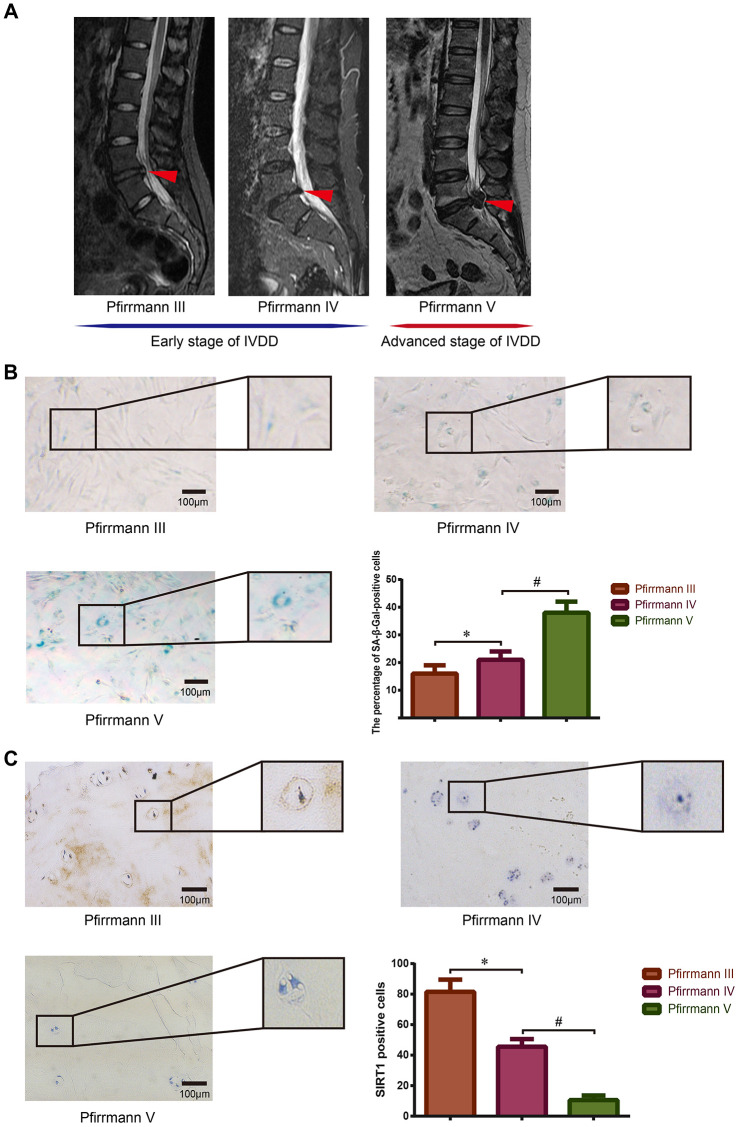
**Detection of SIRT1 expression and cellular senescence in human degenerative NP tissues.** (**A**) Preoperative lumbar MRI images of patients’ IVD tissues were classified via Pfirrmann’s grading system. Degenerative IVDs scored III-IV were defined as early stage of IVDD, and the discs scored V were defined as advanced stage of IVDD. Red arrows refer to the position of the acquired NP tissue. (**B**) The degree of senescence of NP cells isolated from IVDs with different grades of degeneration was detected and analyzed using SA-β-Gal staining (200×). (**C**) The expression level of SIRT1 in NP tissues obtained from patients with different Pfirrmann grades was detected by immunohistochemistry (200×). *P<0.05 versus the Pfirrmann III group. ^#^P<0.05 versus the Pfirrmann IV group. Black bars=100 μm.

First, we demonstrated the relationship between SIRT1 expression and cellular senescence in human NP tissues. The degenerative grades of the lumbar discs from patients undergoing spine surgery were evaluated according to the Pfirrmann classification [35], and representative preoperative magnetic resonance imaging (MRI) scans of donor IVDs with different degrees of degeneration are shown in Figure 1A. SA-β-Gal staining was used to detect the degree of senescence of NP cells derived from NP tissues of patients with different grades of degenerative discs. The results showed that the proportion of SA-β-Gal-positive cells and staining intensity significantly increased with intensified IVDD (Figure 1B). Immunohistochemistry staining of patient NP tissues showed that SIRT1 was expressed in all tissues, whereas the expression level gradually decreased as the disc degeneration intensified (Figure 1C). These results indicated that the expression of SIRT1 was reduced, but the extent of cellular senescence was enhanced as intervertebral disc degeneration progressed.

### High-magnitude compression exacerbated cellular senescence and weakened SIRT1 expression in NP cells

**Figure 2:**
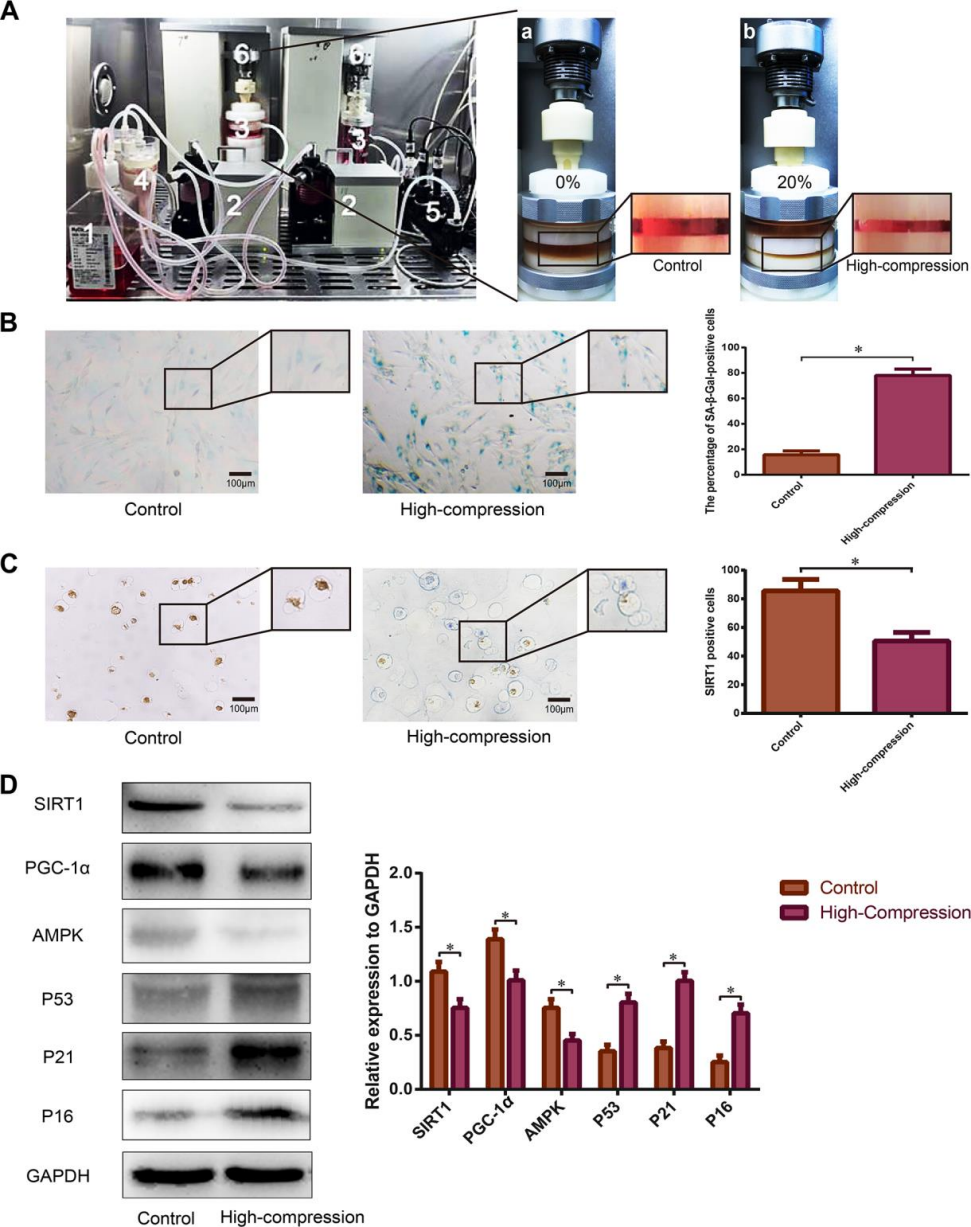
**Analysis of SIRT1 expression and the degree of senescence in human NP cells subjected to high-magnitude compression.** (**A**) Schematic of the primary units of the substance exchanger-based perfusion bioreactor system: (1) medium reservoir; (2) peristaltic pumps; (3) tissue culture chambers; (4) substance exchanger; (5) pH, PO_2_ and PCO_2_ sensor; (6) loading application devices. NP cell-encapsulated Gel-MA hydrogels were dynamically compressed by the loading application devices in the chambers at 0% (a) and 20% (b) compressive deformation for the control and high-compression groups, respectively at a frequency of 1.0 Hz. (**B**) The degree of senescence of the NP cells subjected to 0% or 20% (high-magnitude) compressive deformation was analyzed using SA-β-Gal staining (200×). (**C**) The expression level of SIRT1 in the NP cell-encapsulated hydrogels was detected by immunohistochemistry (200×). (**D**) The SIRT1, PGC1α and senescence-related protein (AMPK, P53, P21 and P16) levels were analyzed by Western blotting. *P<0.05 versus the control group. Black bars=100 μm.

For further analysis of the relationship between SIRT1 expression and cellular senescence in the process of compression-induced NP degeneration, human NP cells seeded in the Gel-MA scaffold were perfusion-cultured with dynamic high-magnitude compression (20% compressive deformation) using our self-developed perfusion bioreactor (Figure 2A). As is shown in Figure 2B, the percentage of SA-β-Gal-positive cells (green), and the staining intensity significantly increased in the NP cells subjected to high-magnitude compression. The expression of senescence-related biomarkers tested by Western blotting revealed that AMPK decreased, whereas P53, P21 and P16 were strongly upregulated in NP cells that experienced high-magnitude compression, which was consistent with results of the SA-β-Gal staining (Figure 2D). The immunohistochemistry data showed that the expression level of SIRT1 substantially decreased in the NP cells under high-magnitude compression (Figure 2C). Additionally, Western blotting results indicated that SIRT1 and PGC-1α were downregulated by high-magnitude compression in human NP cells (Figure 2D); PGC-1α is a marker of the deacetylation activity of SIRT1 [36].

### High-magnitude compression triggered oxidative stress injury and impaired mitochondrial function in NP cells

**Figure 3:**
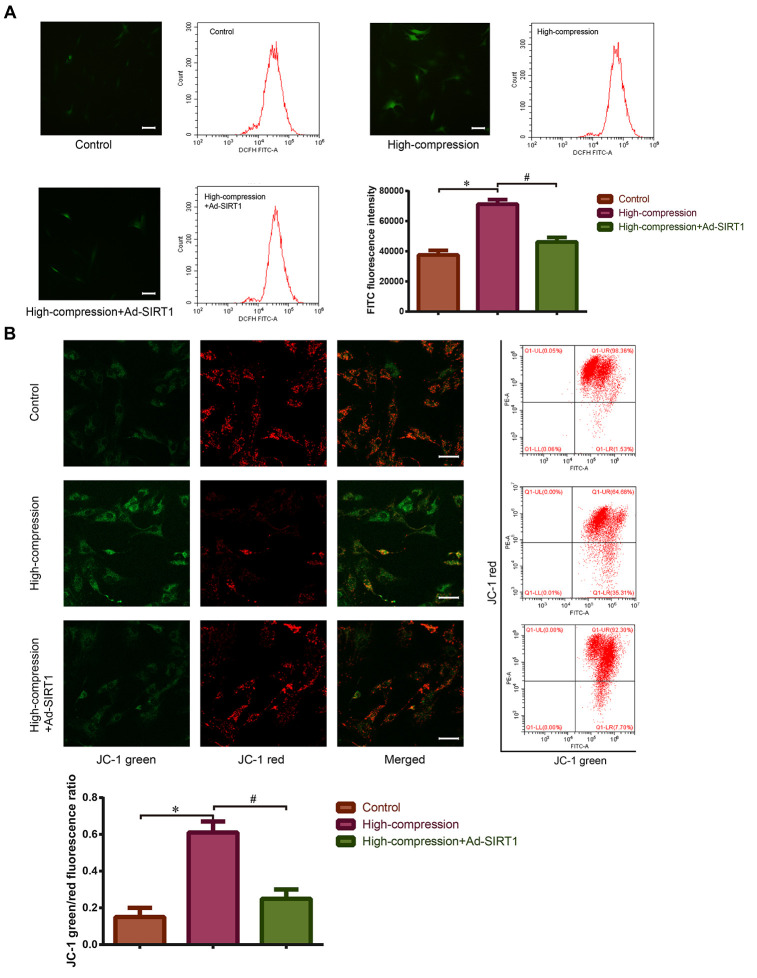
**Measurement of ROS content and mitochondrial membrane potential in the NP cells with or without Ad-SIRT1 treatment under high-magnitude compression.** (**A**) After high-compression or high-compression plus Ad-SIRT1 treatment, the ROS level in NP cells was observed and measured by fluorescence microscopy (200×) and flow cytometry following DCFH-DA fluorescent probe staining. (**B**) The mitochondrial membrane potential of NP cells was observed and measured by fluorescence microscopy (200×) and flow cytometry after JC-1 fluorescent probe staining. The difference in mitochondrial membrane potential is presented by the green/red fluorescence intensity ratio. *P<0.05 versus the control group. ^#^P<0.05 versus the high-compression group. White bars=100 μm.

Our previous study confirmed that high-magnitude compression could accelerate NP cell senescence through the ROS pathway [11]. As the major ROS-generating organelle, mitochondria are also the main target of oxidative stress injury. Thus, we utilized flow cytometry and immunofluorescence to directly and indirectly analyze the content of intracellular ROS in NP cells stained with 2′,7′-dichlorofluorescin diacetate (DCFH-DA) probe, and the data revealed that high-magnitude compression increased the ROS content in NP cells (Figure 3A). Additionally, the mitochondrial membrane potential of the NP cells of each group was detected using JC-1 fluorescent probe staining. The results of immunofluorescence and flow cytometry revealed that the green/red fluorescence ratio of NP cells treated with high-magnitude compression strongly increased, indicating a depressed state of mitochondrial membrane potential (Figure 3B). These data suggested that high-magnitude compression could cause ROS accumulation and trigger mitochondrial dysfunction in NP cells.

### High-magnitude compression caused mitochondrial injury and depressed mitophagy in NP cells

**Figure 4:**
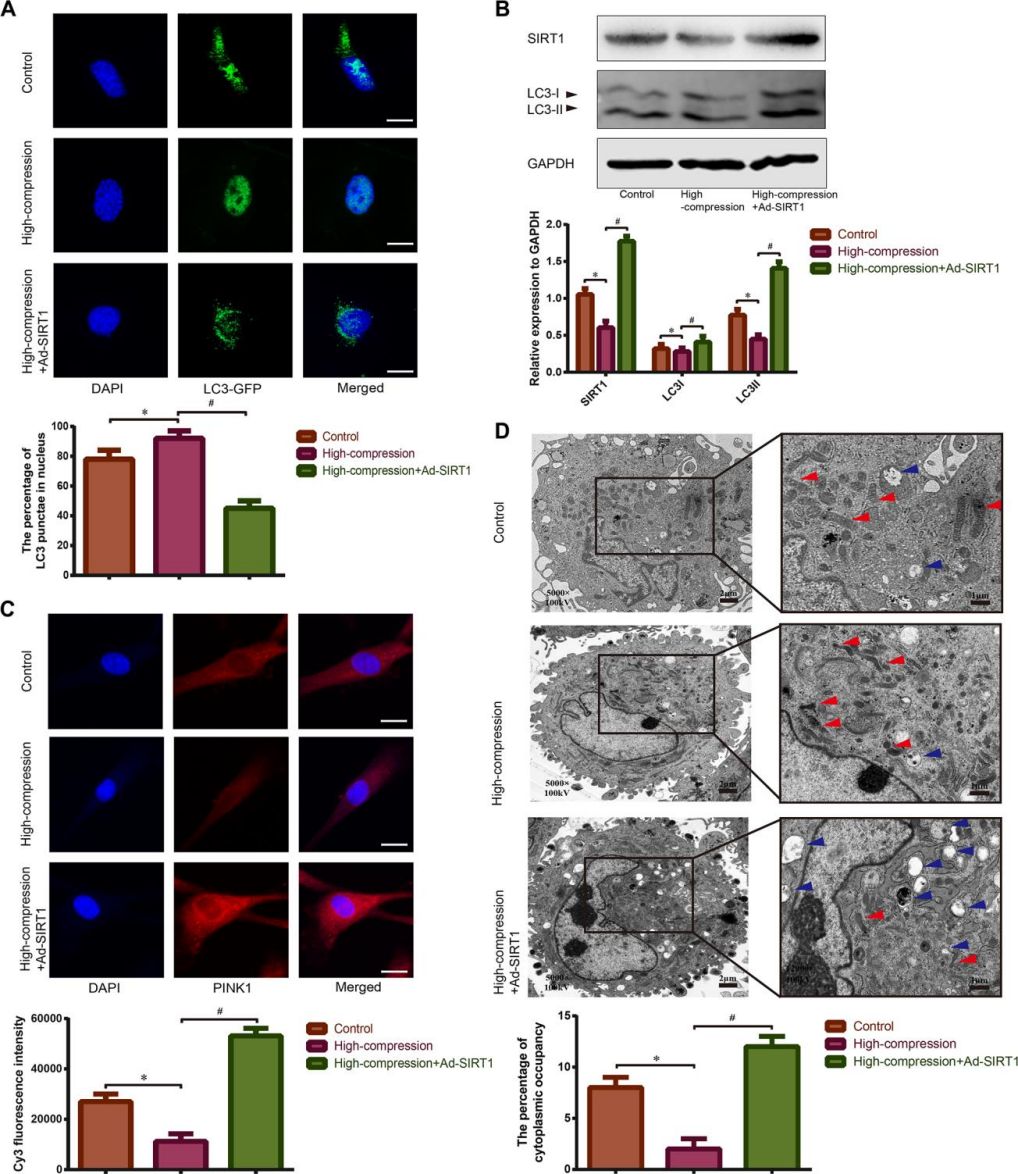
**Observation of mitophagy in human NP cells with or without Ad-SIRT1 treatment under high-magnitude compression.** (**A**) The distribution of GFP-LC3 puncta in NP cells subjected to high-compression or high-compression plus Ad-SIRT1 treatment was visualized by confocal microscopy (400×) after transfection of GFP-LC3-expressing adenovirus. (**B**) The relative expression levels of SIRT1 and LC3II/I in NP cells subject to high-compression or high-compression plus Ad-SIRT1 treatment were analyzed by Western blotting. (**C**) The expression of the key mitophagic regulator PINK1 was observed and analyzed by fluorescence microscopy (400×) after immunofluorescence staining. (**D**) The electron micrographs of mitochondria and autophagosomes in NP cells were observed using TEM (5000×), and double-membrane profiles resembling pieces of mitochondria could be found in some autophagosomes in the Ad-SIRT1-treated group. Blue arrows show the typical autophagic vacuole double-layer ultrastructural morphology. Red arrows represent the electron micrographs of mitochondria in the NP cells from each group. *P<0.05 versus the control group. ^#^P<0.05 versus the high-compression group. White bars=100 μm. Black bars=2 μm.

Mitophagy is found to be a type of mitochondria-specialized autophagy that can selectively remove injured mitochondria to maintain mitochondrial homeostasis [37]. Hence, we hypothesized that there may be a connection between compression-induced mitochondrial dysfunction and mitophagy in NP cells. As is shown in Figure 4A, the NP cells subjected to the high-compression conditions revealed an increase in the amount of nuclear GFP-LC3 puncta, which is a sign of inhibited autophagy. In addition, results of Western blotting also revealed that the expression of SIRT1 and the ratio of LC-3II/I decreased in the high-compression-treated group, indicating that autophagy was retarded (Figure 4B). In addition, an immunofluorescence assay was used to measure the expression level of the mitophagic marker PINK1, and an obvious inhibition of PINK1 expression was found in NP cells subjected to high-magnitude compression (Figure 4C). Furthermore, TEM observation of the morphology of mitochondrial revealed that mitochondria in the compression-treated NP cells appeared smaller than normal with increased membrane density compared with those in the normal NP cells (Figure 4D). In addition, TEM observation of autophagosomes, smooth vacuoles surrounded by a double-membrane without ribosomes, revealed that high-magnitude compression-treated NP cells showed a decreased number of autophagosomes (Figure 4D). The data above further proved that high-magnitude compression could lead to mitochondrial injury, which may involve abnormal mitophagy.

### Upregulation of SIRT1 activated mitophagy and alleviated mitochondrial injury in human NP cells under high-magnitude compression.

Previously, we speculated that SIRT1 may protect against compression-induced senescence in NP cells by regulating mitophagy, due to its antioxidant and mitophagy-acting properties. For confirmation of this hypothesis, SIRT1 was upregulated using Ad-SIRT1, and the status of mitophagy and mitochondrial function in NP cells subjected to high-magnitude compression were analyzed. As is shown in Figure 3A, 3B, treatment with Ad-SIRT1 significantly attenuated the ROS content and alleviated the abnormal state of mitochondrial membrane potential in the NP cells subjected to high-compression. Notably, in NP cells, the switch to high-compression conditions resulted in an increase in the amount of nuclear GFP-LC3 puncta, whereas distribution of GFP-LC3 translocated from the nucleus into the cytoplasm when the NP cells were treated with Ad-SIRT1 (Figure 4A). Moreover, Western blotting analysis and immunofluorescence observation showed enhanced expression levels of SIRT1, LC3-II/I and PINK1 in the SIRT1-overexpressing NP cells compared with the other groups of cells (Figure 4B, 4C). Additionally, TEM observation showed a substantially increased number of autophagosomes in the Ad-SIRT1-treated NP cells compared with the NP cells from the control and high-compression-treated groups (Figure 4D). TEM observation also found fragmentations that resemble mitochondrial sections being engulfed by double-layer vesicles in Ad-SIRT1-treated NP cells, which is an indicator of mitophagy (Figure 4D).

### Blocking of mitophagy attenuated the anti-senescence role of SIRT1 in NP cells under high-magnitude compression.

**Figure 5:**
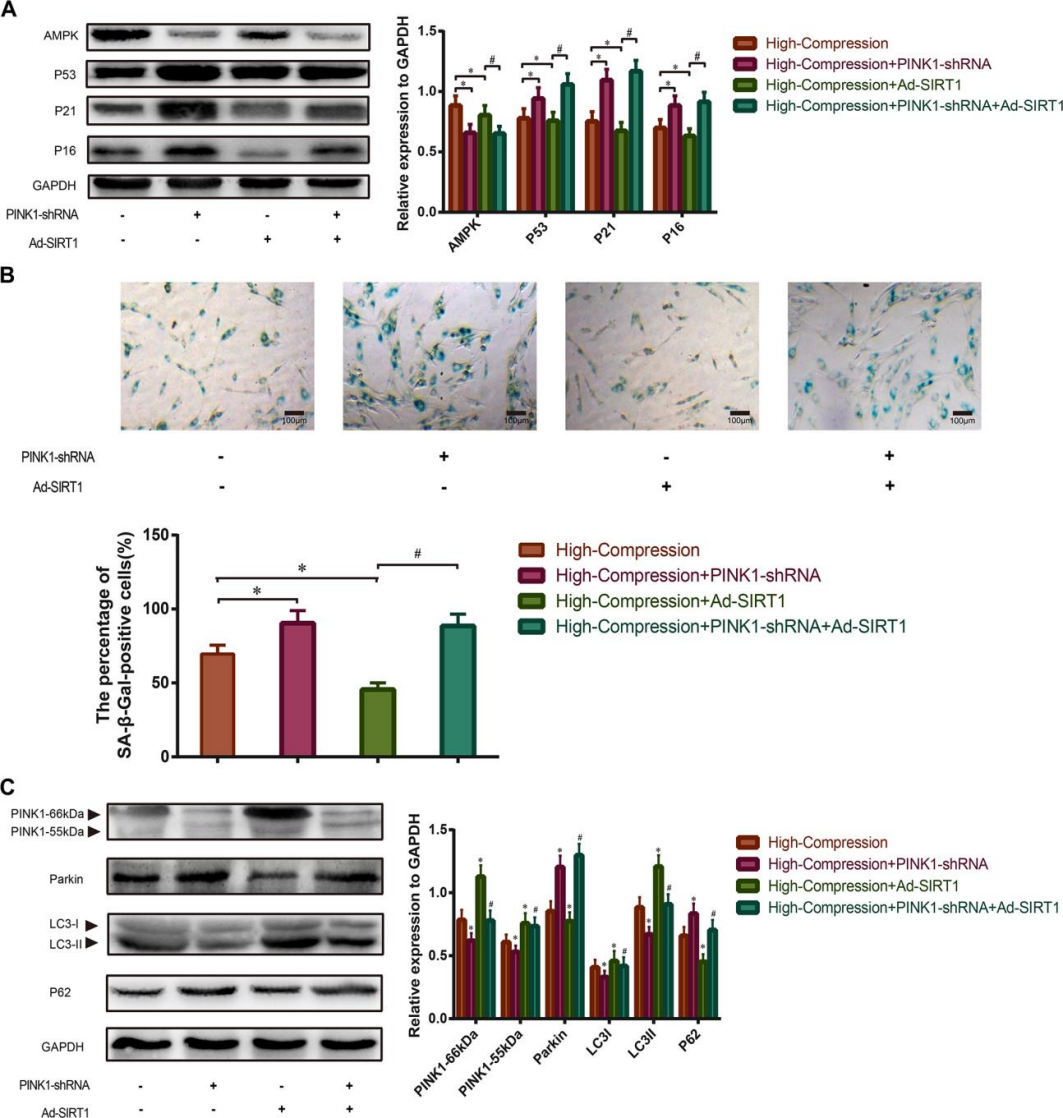
**Effect of SIRT1-overexpression on high-compression treated NP cells under inhibition of mitophagy.** (**A**) PINK1-shRNA was used to silence the endogenous PINK1 in NP cells before high-compression and Ad-SIRT1 treatments. The expression levels of senescence-related biomarkers (AMPK, P53, P21 and P16) in each group were analyzed by Western blotting. (**B**) The degree of senescence of the NP cells in each group was assessed by optical microscopy after SA-β-Gal staining (200×). (**C**) The expression levels of mitophagic biomarkers (PINK1, Parkin, LC3II/I and P62) were analyzed by Western blotting. *P<0.05 versus the high-compression group. ^#^P<0.05 versus the high-compression+Ad-SIRT1 group. Black bars=100 μm.

To further clarify that SIRT1 protects against compression-induced senescence of NP cells by inducing mitophagy, we transfected NP cells with PINK1-shRNA to silence the endogenous PINK1 expression before compression treatment. Next, the expression levels of senescence-related proteins (AMPK, P53, P21, P16) were analyzed using Western blotting, and the data showed that the expression levels of AMPK decreased, whereas P53, P21 and P16 increased in the high-magnitude compression+PINK1-shRNA-treated group (Figure 5A). In addition, administration of Ad-SIRT1 significantly enhanced the expression level of AMPK and attenuated the expression of P53, P21 and P16 in the NP cells under high-magnitude compression, but this treatment did not have the same effect in the PINK1-knockout NP cells (Figure 5A). Moreover, the results of SA-β-Gal staining also revealed that the NP cells treated with high-magnitude compression following administration of PINK1-shRNA showed the highest proportion of positively stained cells. In contrast, the Ad-SIRT1-treated NP cells had the lowest proportion of SA-β-Gal-positive cells as well as the weakest staining intensity (Figure 5B). Nevertheless, knockout of PINK1 inhabited the protective role of SIRT1 against high-magnitude compression-induced senescence in NP cells, which is consistent with the Western blotting results (Figure 5B). Next, we further analyzed the expression differences of mitophagic proteins in each group by using Western blotting. The results revealed that the expression of PINK1 and LC-3 II/I was strongly attenuated, but the expression of Parkin and P62 was enhanced in the NP cells treated with PINK1-shRNA transfection, which indicated that mitophagy was inhibited in the PINK1-knockout NP cells (Figure 5C). Furthermore, treatment with Ad-SIRT1 enhanced the expression of PINK1 and LC-3 II/I, and reduced the expression of Parkin and P62 in NP cells, indicating enhanced activation of mitophagy (Figure 5C). However, when PINK1 was knocked out in advance, the mitophagy-stimulating role of SIRT1 was also attenuated (Figure 5C). These results implied that SIRT1 could alleviate high-magnitude compression induced senescence of human NP cells by activating mitophagy.

## DISCUSSION

IVDD is considered to be the most common pathological basis for spine degenerative disease, which is the major etiological factor of low back and leg pain [1]. The progressive degradation of ECM is the main pathological characteristic during IVDD [6]. NP cells are responsible for the synthesis of ECM within the NP region of the IVD; hence, the accumulation of senescent NP cells is regarded as a direct reason for the compromised matrix synthesis [3, 4]. However, previous researchers did not find a direct relationship between the patient’s age and senescent cell accumulation [3, 4, 38]. Compared with that in degenerative IVD, the expression of senescence-related biomarkers was not significantly upregulated in normal age-matched IVD [3, 4, 38]. Thus, these findings indirectly suggest that the senescent phenotype in degenerative IVD closely resembles stress-induced senescence caused by extrinsic stresses instead of the intrinsic aging process. Therefore, further investigation of the pathomechanism behind NP cell senescence and the development of strategies to retard the progression of NP cell senescence will be helpful for the treatment of spine degenerative diseases.

Human IVD mainly performs three primary functions: (a) resists compressive and tension forces during movement, (b) provides flexibility to the spine, and (c) maintains space between vertebral bodies, thereby preventing compression or injury to the spinal nerves [39]. Thus, it is understandable that NP tissue suffers various types of mechanical compression under in vivo conditions. Established studies have demonstrated that un-physiological and high compressive stress can aggravate NP cell death and exacerbate disc degeneration in animal model [8, 40]. Hence, we speculated that inhibition of high compressive stress-induced senescence of NP cells may play a vital role in retarding IVDD. Previously, we have demonstrated that high-magnitude compression could accelerate the premature senescence of NP cells, and the mechanism involved in this process was associated with oxidative stress and the ROS pathway [11]. As the major regulator of ROS generation and redox homeostasis, mitochondrial have been widely reported to be associated with NP degeneration [18, 19]. Thus, in the present study, we further detected the mitochondrial function of the NP cells subjected to high-magnitude compression by analyzing ROS content, measuring mitochondrial membrane potential and observing mitochondrial micromorphology under TEM. The experimental results indicated that high-magnitude compression caused ROS accumulation and induced mitochondrial dysfunction in NP cells.

SIRT1 has been widely reported to play a protective role against NP degeneration via multiple pathways [25, 26, 41]. The present research also revealed that the expression of SIRT1 in clinical NP samples gradually decreased, whereas the extent of cellular senescence was enhanced as disc degeneration intensified. Moreover, upregulation of SIRT1 substantially alleviated the content of intracellular ROS content and senescence of NP cells that were perfusion-cultured and underwent high-magnitude compression in vitro. Recent studies have noted that SIRT1 performs its anti-senescence and antioxidant functions by regulating mitophagy, which is a mitochondrion-protective process [27–30]. Our experimental data indicated that high-magnitude compression depressed the mitochondrial membrane potential in NP cells, whereas upregulation of SIRT1 considerably elevated the mitochondrial membrane potential to the normal state in NP cells subjected to high-magnitude compression. In fact, the variety of mitochondrial membrane potentials indirectly reflects the activation of mitophagy [42]. Mitophagy is a type of mitochondria-targeted autophagy that ameliorates various types of stress-induced cellular damage by recycling injured mitochondria [37, 43]. Additionally, established studies have verified the beneficial role of mitophagy in NP cell survival [33, 34]. Notably, another study also elucidated the relationship between SIRT1-induced autophagy and nutrient deprivation-induced mitochondrial apoptosis of NP cells [44]. Additionally, as an NAD-dependent deacetylase, SIRT1 could affect mitophagy via deacetylation of the key autophagic protein LC3 in the nucleus [28, 45]. In view of the published studies and our experimental data, we further speculated that SIRT1 exerted beneficial effects through attenuation of compression-induced senescence by regulating mitophagy. To verify this hypothesis, we used PINK1-shRNA to silence the endogenous PINK1 expression before compression and SIRT1-overexpressing treatment, because the PINK1-Parkin axis is regarded as the core machinery for mitophagy [46]. Then, the degree of senescence and mitophagic activity in NP cells were analyzed. Analysis of the senescent cells and senescence-related biomarkers suggested that deletion of PINK1 substantially exacerbated senescence in NP cells under compression stress, whereas treatment with Ad-SIRT1 effectively attenuated the compression stress-induced senescence of NP cells. However, SIRT1-overexpressing did not reverse the degree of senescence in the PINK1-knockout NP cells suffering high-magnitude compression, indicating that the lack of PINK1 blocked the protective property of SIRT1 against compression stress-induced senescence in NP cells. Furthermore, analysis of mitophagy-related proteins revealed that treatment with Ad-SIRT1 significantly enhanced the expression of PINK1 and an autophagic marker (LC3II/I), but decreased the expression levels of Parkin and P62. This finding can be explained as follows: when the process of mitophagy is activated, full-length PINK1 (66 kDa) is modified into a cleaved product (55 kDa), which translocates on the outer surface of the targeted mitochondrion and recruits the Parkin from the cytosol [43, 46]. Recruited Parkin protein combines with LC3II and then initiates the process of mitophagy to clear the targeted mitochondrion [46]. Nevertheless, in the normal mitochondrion, full-length PINK1 is released into the cytosol and then degraded by mitochondrial proteases and proteasomes [43, 46]. TEM observation also found a markedly increased number of autophagosomes and signs of mitophagy (autophagosome-engulfed mitochondrial sections) in the Ad-SIRT1-treated NP cells. Therefore, our study concludes that SIRT1 can alleviate mechanical stress-induced senescence of human NP cells under high-magnitude compression by activating mitophagy.

**Figure 6:**
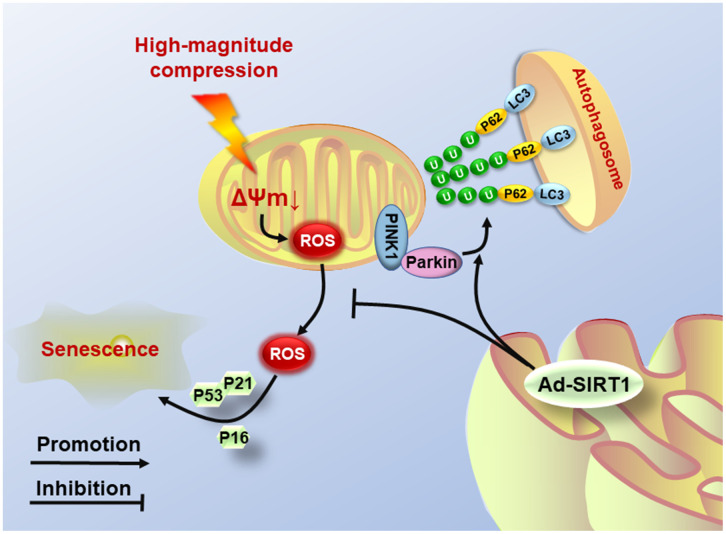
**Schematic diagram shows the potential mechanism of SIRT1 overexpression.** High-magnitude compression triggered mitochondrial dysfunction and induced NP cell senescence. Overexpression of SIRT1 could alleviate compression-induced senescence by activating mitophagy to eliminate injured mitochondria and retard ROS accumulation.

Although there are some novel findings in the present study, several limitations also exist. First, the NP cells used in the study were cultured under normoxic conditions, because of the lack of hypoxia-culture settings in our bioreactor. This condition is different from the physiological hypoxic condition in which the NP cells live. If possible, we will update the equipment to obtain similar hypoxic microenvironments in future studies. Second, autophagy is a quite complex and controversial process in regulating cell survival, and the present study also found that SIRT1 deacetylase could regulate the autophagy level, and knockout of PINK1 did not completely inhibit the activation of autophagy in NP cells. Thus, further research on the SIRT1-induced autophagy involved in the pathomechanism of NP degeneration is needed in the future. To conclude, our study reveals that SIRT1 expression is associated with the progression of IVDD and is also involved in the regulation of mechanical stress-induced damage to the mitochondrial and senescence in human NP cells. Moreover, upregulation of SIRT1 can effectively suppress the generation of ROS and protect against senescence in NP cells subjected to high-magnitude compression by recycling injured mitochondria by mitophagy (Figure 6). This study helps us better understand the regulatory role of SIRT1 in the process of NP cell senescence and provides more substantial evidence for the usage of SIRT1-targeting agents in the treatment of IVDD.

## MATERIALS AND METHODS

### NP tissue collection

The experimental subjects of this study were obtained from spine surgery patients admitted to the orthopedics department of the Third Affiliated Hospital of Chongqing Medical University. The Pfirrmann classification system was used to define the degeneration grades of IVD by preoperative MRI scans [35]. Degenerative IVDs scored III-IV were defined as early stage of IVDD, and the discs scored V were defined as advanced stage of IVDD. All the experimental samples were obtained from lumbar spine surgery patients (mean age: 45 years; age range: 37-52 years; Pfirrmann III-IV: 6 males, 5 females; Pfirrmann V: 5 males, 5 females). Informed consent was obtained from all patients involved in our study, and our research plan complied with approval from the ethics committee of Chongqing Medical University.

### Isolation and culture of primary NP cells

According to our previous study, primary NP cells from surgery patients were isolated and cultured as follows [34]. In brief, NP tissues of the excisional disc were gathered with tweezers and minced into flocculent pieces under sterile conditions. Then, the isolated tissue pieces were digested in 0.2% type II collagenase (Sigma, USA) for approximately 4 h, following 0.25% trypsin solution (Sigma, USA) digestion at 37°C for 20-40 min. After that, complete culture medium (DMEM/F-12 (Gibco, USA) containing 15% fetal bovine serum (FBS, Gibco, USA) and 1% penicillin/streptomycin (Sigma, USA)) was utilized to neutralize the type II collagenase. The suspension was then centrifuged at 1500 rpm for 10 min to deposit the NP cells. Next, cells were resuspended and seeded into flasks with 5 ml complete culture medium at 37°C in an atmosphere containing 5% CO_2_. And when cellular confluence grew to 80-90%, NP cells were trypsinized with 0.25% trypsin (Sigma, USA) for subculture. All experiments were carried out with NP cells at passage II.

### Bioreactor culture and compressive loading application

The Gel-MA photocrosslinking hydrogels used as the scaffold for NP cell perfusion-culture in the bioreactor were freely provided by the Tissue Engineering Center of Third Military Medical University. And the cell-seeding procedure was performed according to our previous study [10]. Briefly, NP cells at passage II were trypsinized with 0.25% trypsin (Sigma, USA), and the suspension was then centrifuged at 1500 rpm for 10 min to deposit the cells. Next, the collected NP cell pellets were premixed with the liquid Gel-MA hydrogel at a density of 1×10^7^ cells/mL. Then, the cell-hydrogel mixture was injected into a cylindrical mold (Ø=5 mm, height=5 mm) and exposed to 365 nm ultraviolet light for 1 min to construct the network. Then, the elastic cell-encapsulated hydrogels were precultured in the culture dish in 5% CO_2_ at 37°C for 2 days. After that, the NP cell-encapsulated hydrogels were placed into the tissue culture chambers of the self-developed substance exchanger-based perfusion bioreactor system (Figure 2A) and perfusion-cultured for 5 days. Simultaneously, NP cell-encapsulated hydrogels were dynamically compressed by the loading application devices in the chambers at 0% and 20% compressive deformation for the control and high-compression groups, respectively, at a frequency of 1.0 Hz for 4 h once per day. The compressed NP cells used for subsequent experiments were washed out from the hydrogel scaffold after digestion with 0.2% type II collagenase (Sigma, USA) at 37°C for 3-5 min.

### NP cell transfections

The recombinant lentiviral vectors (Ad-SIRT1 and PINK1-shRNA) were purchased from GeneChem (Shanghai, China). The NP cells were transfected with the lentiviral vector according to the manufacturer's protocol. Briefly, after reaching 30-50% confluence, the NP cells were transfected with lentivirus at a multiplicity of infection (MOI) of 50. After 12 h of transfection, more than 95% of the cells were still alive. The culture medium was discarded, and the medium was replaced with fresh complete culture medium. Three days later, the transfected NP cells were subcultured into the scaffolds for the subsequent treatments and experiments.

### SA-β-Gal staining

SA-β-Gal staining was used to detect the degree of senescence of NP cells and performed according to the protocol of the SA-β-Gal kit (Beyotime, China). Briefly, after being washed with PBS buffer twice, NP cells (1×10^6^/well/group) on 6-well plates were fixed with 0.2% paraformaldehyde for 20 min at room temperature. Next, the treated cells were stained with X-gal staining solution overnight at 37°C. Images were taken by optical microscopy (Olympus, Japan) and the percentages of SA-β-gal-positive cells were determined for statistical analysis.

### Immunohistochemical staining

An immunohistochemical staining assay (Boster Biological Technology, China) was used to evaluate the protein deposition and expression level of SIRT1 in human NP tissues and NP cell-encapsulated hydrogels following the manufacturer's protocol. Briefly, the harvested IVDs and hydrogels were sequentially paraformaldehyde-fixed for 24 h, paraffin-embedded and sectioned at 4 mm. Next, the sections were treated with 3% H_2_O_2_ for 15 min at room temperature to eliminate endogenous peroxidase activity and were subsequently incubated with 0.125% trypsin for 30 min at 37°C to retrieve the antigen, before being blocked with normal goat serum for 15 min at room temperature. The sections were incubated with rabbit anti-SIRT1 (1:200; Cell Signaling, USA) primary antibodies overnight at 4°C. Then, the sections were incubated with goat anti-rabbit IgG-HRP secondary antibody (1:1000; Proteintech, China) and counterstained with hematoxylin. The resulting sections were photographed under a microscopy (Olympus, Japan). Five fields which distributed throughout the whole section were counted (200×), and the average positive rate was counted and statistically analyzed.

### Immunofluorescence staining

The harvested NP cells were fixed with 4% paraformaldehyde for 10 min and then treated with 5% Triton for 5 min. Then, the cells were stained with 1 μg/ml rabbit anti-PINK1 (1:200; Abcam, USA), a mitophagy-related marker antibody overnight at 4°C. Next, the cells were incubated with the anti-rabbit fluorescent secondary antibody (1:1000; Proteintech, China) at room temperature for 2 h. The cells were then counterstained with 4',6-diamidino-2-phenylindole (DAPI) and imaged using a fluorescence microscopy (Leica, Germany).

### Examination of mitochondrial membrane potential

JC-1 probe staining (Beyotime, China) was utilized to detect the mitochondrial membrane potential of NP cells. When the mitochondrial membrane potential of cells is maintained in a normal state, the JC-1 probe aggregates in the mitochondrial matrix and emits red fluorescence. When the mitochondrial membrane potential is reduced, the JC-1 probe cannot aggregate in the mitochondrial matrix and emits green fluorescence [42]. Thus, the green/red fluorescence intensity ratio can indirectly reflect the state of the mitochondrion. The staining was performed according to the manufacturer's protocol. In brief, the treated cells were seeded into the 6-well plates and cultured for 24 h. Then, after the cells were washed with PBS buffer for three times, 1 ml of complete culture medium was added to each plate. Next, the cells were immersed in 1 ml JC-1 working solution and incubated at 37°C for 20 min. After the cells were washed with cold JC-1 staining buffer for three times, the mitochondrial membrane potential of the cells was detected by fluorescence microscopy (Leica, Germany), and quantitated using a flow cytometer (Beckman Coulter, USA) after being collected. Finally, the green/red fluorescence intensity ratio was statistically analyzed.

### Measurement of intracellular ROS content

The level of intracellular ROS was assessed using DCFH-DA probe staining (Beyotime, China). In brief, treated cells were seeded in 6-well plates and cultured with complete culture medium for 24 h. Next, 10 mg/ml DCFH-DA was added to 1 ml DMEM/F12, and the cells were incubated for 20 min at 37°C. Then, the cells were observed under a fluorescence microscopy, and quantitated by using a flow cytometer (Beckman Coulter, USA) at an emission wavelength of 525 nm and an excitation wavelength of 488 nm after being collected.

### GFP-LC3 analysis

For observation of the autophagosomes, NP cells were transfected with a GFP-LC3-expressing adenovirus for 24 h. Then, the transfected cells were used for the subsequent experiments. The autophagosomes were visualized through laser confocal microscopy (Leica, Germany) and the level of autophagy was evaluated with the number of green fluorescent puncta of autophagosomes.

### Protein extraction and Western blotting

In brief, after being washed with cold PBS for 3 times, NP cells were lysed with RIPA lysis buffer containing 1% PMSF (Beyotime, China) for 30 min at 4°C. Then, the whole-cell lysates were centrifuged at 12000×g for 10 min at 4°C. Next, an enhanced bicinchoninic acid (BCA) kit (Beyotime, China) was used to detect the concentration of the obtained protein. Then, the obtained protein samples were subjected to 12% SDS-polyacrylamide gel electrophoresis (SDS-PAGE) and transferred by electroblotting to PVDF membranes (Millipore, USA). Then, the bands were blocked with 5% nonfat dry milk in TBST (1% Tween 20 in TBS) for 40 min at room temperature, and then incubated with the primary antibodies (anti-SIRT1, anti-Parkin (1:1000; Cell Signaling, USA), anti-LC3, anti-P62, anti-PINK1 (1:1000; Abcam, USA), anti-AMPK, anti-PGC1α, anti-P53, anti-P21, anti-P16 and anti-GAPDH (1:500; Proteintech, China)) overnight at 4°C. After the blots were washed with cold TBST for 3 times, they were incubated with the secondary antibody for 80 min at room temperature. Finally, after the blots were washed with cold TBST for 3 times, the intensity of the blots was detected by Image Lab software (Bio-Rad, USA).

### TEM observation

After being trypsinized and collected into a pellet, NP cells were immersed in buffer containing 2.5% glutaraldehyde, and 2.0% paraformaldehyde in 0.1 M sodium cacodylate buffer (pH 7.4) overnight at room temperature. Then, the samples were washed with 0.1 M phosphate-buffered saline and then fixed in 2.0% osmium tetroxide for 1 h at room temperature. After being washed again in buffer followed by dH_2_O, and dehydrated via an ascending ethanol series, the samples were embedded in EMbed-812. Then, the samples were cut into thin sections and stained with lead citrate. Finally, the treated samples were observed using a transmission electron microscope (Hitachi, Japan) and images were captured using AMT Advantage software.

### Statistical analysis

All quantitative assays were performed in triplicate, and the data are expressed as the mean ± standard deviation. The data were analyzed using SPSS version 20.0 software (SPSS, Inc., USA). One-way analysis of variance (ANOVA, p<0.05) was used to identify statistically significant differences among multiple groups. Unpaired t-tests (p<0. 05) were used to analyze individual groups.
